# Characterization of Transgenic Silkworm Yielded Biomaterials with Calcium-Binding Activity

**DOI:** 10.1371/journal.pone.0159111

**Published:** 2016-07-14

**Authors:** Shaohua Wang, Yuyu Zhang, Mingying Yang, Lupeng Ye, Lu Gong, Qiujie Qian, Yajun Shuai, Zhengying You, Yuyin Chen, Boxiong Zhong

**Affiliations:** 1 College of Animal Sciences, Zhejiang University, Hangzhou 310058, P.R. China; 2 College of Life Sciences, Zhejiang University, Hangzhou 310058, P.R. China; Institute of Plant Physiology and Ecology, CHINA

## Abstract

Silk fibers have many inherent properties that are suitable for their use in biomaterials. In this study, the silk fibroin was genetically modified by including a Ca-binding sequence, [(AGSGAG)_6_ASEYDYDDDSDDDDEWD]_2_ from shell nacreous matrix protein. It can be produced as fibers by transgenic silkworm. The Ca-binding activity and mineralization of the transgenic silk fibroin were examined in vitro. The results showed that this transgenic silk fibroin had relatively higher Ca-binding activity than unmodified silk fibroin. The increased Ca-binding activity could promote the usage of silk fibroin as a biomaterial in the pharmaceutical industry. This study shows the possibility of using silk fibroin as a mineralization accelerating medical material by generating genetically modified transgenic silkworm.

## Introduction

The silkworm *Bombyx mori* has been used as bioreactor to produce foreign proteins for decades due to its advanced physiological characteristics, such as short life cycle, convenient breeding process, large-scale protein production, and small individuals, which can be maintained in high densities, especially after the germline transformation method for the silkworm was developed using the transposon *piggyBac*[1–4]. Silk produced by silkworm is a natural protein fiber that contains two main components: fibroin and sericin. Fibroin is synthesized and secreted in the posterior silk gland, coated by sericin when it accumulates in the lumen of the middle silk gland and secreted into the cocoon via the anterior silk gland[5]. Three proteins, a 350 kDa fibroin heavy chain, a 26 kDa fibroin light chain and P25/fibrohexamerin, compose the silk fibroin[6].

The clinical need of materials for bone regeneration is expected to increase, and some essential characteristics of these materials, including biocompatibility, porosity, and appropriate mechanical properties, are important for their application. The silk fibroin from silkworm, with superior mechanical properties such as the ability to be tailored, slow degradation, adequate time permitted for remodeling, and, most importantly, biocompatibility, is an ideal biomaterial for clinical uses. The suture made from the silk fibroin has been used for decades[7]. Sponge made from silk fibroin has been used as a scaffold for chondrocyte distribution and cartilage regeneration; similarly, silk can be used as a Nano carrier for drug delivery or as a ligament to regenerate human bone tissues [8–10]. In addition, cell-adhesive activity increases when the surfaces of plates for mammalian cell cultures are coated by silk fibroin with incorporation of collagen- or fibronectin-derived peptides produced by transgenic silkworm[11]. Bone repair has been explored based on calcium binding silk scaffolds using transgenic silk fibroin produced by transgenic silkworm with Ca-binding sequence [(AGSGAG)_4_E_8_AS]_4_ [12].

The protein [(AGSGAG)_3_AS(AGSGAG)_3_ASEYDYDDDSDDDDEWD]_2_ purified from *E*. *coli* was reported to have the ability to bind calcium ions under the certain conditions and can be dip-coated with hydroxyapatite[13]. This peptide sequence was combined with the calcium binding site EYDYDDDSDDDDEWD from the pearl oyster (*Pinctada fucata*) nacreous layer matrix protein MSI60[14] and the *B*. *mori* silk fibroin repetitive domain (AGSGAG)_n_. In the present study, the transgenic silk fibroin containing the Ca-binding sequence [(AGSGAG)_6_ASEYDYDDDSDDDDEWD]_2_ (referred to as CABP) was produced as transgenic silk fibers through the systematic transformation of silkworm. The Ca-binding activity and mineralization of the transgenic silk fibroin was examined in vitro. This silk fibroin-based biomaterial with Ca-binding activity can be produced in large scale by silkworm breeding.

## Materials and Methods

### Construction of the Ca-binding protein expression vector

**Fig 1:**
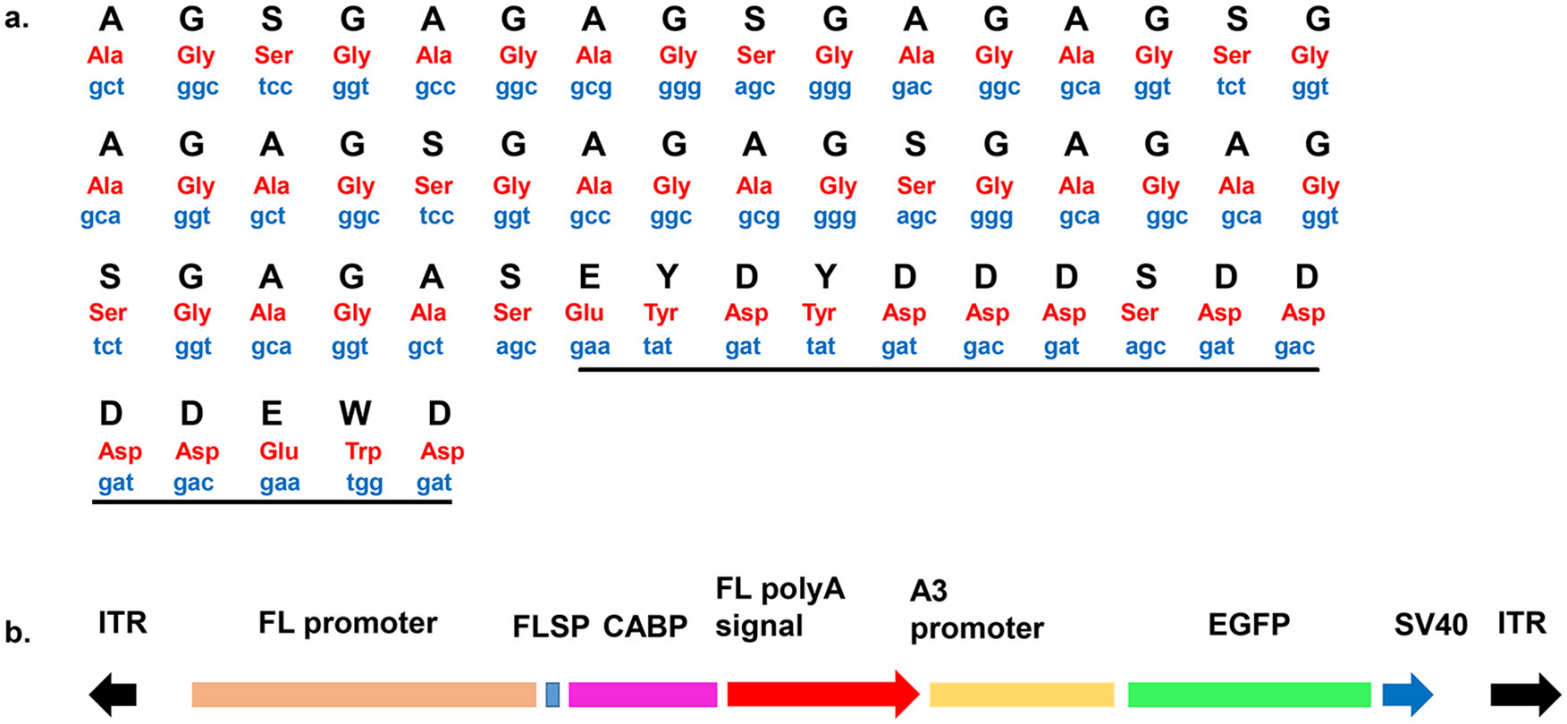
The calcium binding protein sequence and transgenic experiment vector. One copy of the sequence [(AGSGAG)_6_ASEYDYDDDSDDDDEWD]_2_ (A). (AGSGAG)_n_ is the silk fibroin repetitive domain from *B*. *mori*. The calcium-binding protein sequence from the shell nacreous matrix protein MSI60 is marked by a black line. The vector used for the transgenic experiment (B). ITR, inverted terminal repeats of PB transposon; FL, fibroin light chain; FLSP, signal peptide of fibroin light chain; CABP, calcium-binding protein; A3, *Bombyx mori* A3 cytoplasmic actin gene; EGFP, enhanced green fluorescence protein; SV40, 3’-untranslated sequences.

Dimerized DNA fragments containing the CABP sequence with certain improvements according to a report described previously[15] were used in this study. The sequence was artificially synthesized by GenScript (Piscataway, NJ, USA) (Fig 1a). The plasmid constructed for Ca-binding protein expression was based on the transposon plasmid pBA3EGFP maintained by our lab. The vector contains two expression frames. One of them is used for CABP expression and contains the fibroin light chain promoter (BGIBMGA009393, from -4021 to -1) (FLP), fibroin light chain signal peptide (FLSP), CABP, and fibroin light chain 3’-flank (BGIBMGA009393, from +12793 to +13299). The other is used for expression of the reporter gene enhanced green fluorescent protein (EGFP) and is composed of the *Bombyx mori* A3 cytoplasmic actin gene promoter (A3), EGFP, and 3’-untranslated sequences (SV40). Genomic DNA was extracted from silk gland of Qiufeng silkworm strain using a DNA extraction kit (Sangon, Shanghai, China). The fibroin light chain promoter and 3’-flanking region were amplified using high fidelity PrimeSTAR HS DNA Polymerase (TAKARA BIO INC., Otsu, Shiga, Japan). Two clones were sequenced after cloning into the pMD19-T vector (TAKARA BIO INC., Otsu, Shiga, Japan). The signal peptide sequence was synthesized by combining it to the primer used to get fibroin light chain promoter. The structure of the final plasmid used for microinjection is *piggyBac*-FLP-FLSP-CABP-FL-3’polyA+A3-EGFP-SV40, which was named pBCABP-EGFP (Fig 1b). The produced protein sequence is [(AGSGAG)_6_ASEYDYDDDSDDDDEWD]_2_, and the molecular weight is predicted as 12.1 kDa by ProtParam tool (http://web.expasy.org/compute_pi/).

### Embryo injection and positive individual screening

The methodologies for embryo microinjection and positive animal screening have been described in detail previously[16]. Eggs of polyvoltine strain Lan10 were harvested at the syncytial preblastoderm stage between 3 and 4 h after laying at 25°C. Then, 5–10 nl of the mixture of pBCABP-EGFP and helper plasmids (1:1, total DNA concentration being 0.4 μg/μl) was microinjected into each egg. The helper plasmids were kindly provided by Dr. T. TAMURA (National Institute of Sericultural and Entomological Science, Tsukuba, Japan). The embryos were allowed to develop at 25°C with the humidity maintained at 85%. The EGFP reporter gene was used to screen the positive individuals at 1^st^ instar the lava period.

### Inverse PCR analysis

Genomic DNA was extracted from positive silkworm individuals using a DNA extraction kit (Sangon, Shanghai, China). Then, inverse PCR analysis was conducted as previously described[17]. Briefly, 1 μg of genomic DNA was digested by Sau3A I at 37°C for 2 h and then self-circularized overnight at 16°C by T4 DNA ligase (TAKARA BIO INC., Otsu, Shiga, Japan). The ligated products were amplified using EX Taq polymerase (TAKARA BIO INC.) and primers with 2 min denaturing at 94°C, followed by 35 cycles of 30 s at 94°C, 30 s at 58°C, and 2 min at 72°C, and a final extension at 72°C for 10 min. The primers used for the insertion site identification were L1-1: GACAAGCACGCCTCAGCC, L1-2: GATAATCATGCGTCATTTTGACTCA, L2-1: GCTCCAAGCGGCGACTG, and L2-2: ATGCTCATCGTCTAAAGAAC. Amplified products were sequenced after cloning into the pMD19-T (TAKARA BIO INC.) vector to determine the insertion sites in the chromosome.

### Western Blot

The posterior silk gland of the transgenic silkworm and its donor strain at 3^rd^ day of the 5th instar were isolated according to the method previously described[18]. Briefly, the posterior silk glands were isolated and washed at 4°C with 0.7% NaCl. The cleaned glands were immersed in 60% ethanol for 2 min. The cells of the posterior silk gland and the silk coated by these cells were separated for protein extraction. Then, 10 μl sample extraction buffer (glycerol 10ml, 0.5M Tris-HCl (pH 6.8) 12.5ml, SDS 2.5g, ß-mercaptoethanol 5ml, add water to 100ml) for SDS-PAGE was added to 1 mg of tissue and ground using a pestle. The resultant suspension was centrifuged twice at 4°C, 15000 rpm for 10 min. The supernatant was stored at -20°C for later use. The western blot experiments were performed according to the procedures described previously [19]. SDS-PAGE was performed using a 15% polyacrylamide gel, and the separated proteins were stained by Coomassie Brilliant Blue R-250 (Bio-Rad, Hercules, CA, USA). Blocking of the separated protein was performed using PBST (0.1% (v/v) Tween 20) containing 5% (w/v) skimmed milk and interacted with the antibody designed based on the (AGSGAG)_6_ASEYDYDDDSDDDDEWD by GenScript (Piscataway, NJ, USA). The target site was AGSGAGASEYDYDDC. The first antibody was obtained from New Zealand rabbits. The concentration of the antibody was 1:2000. Goat anti-mouse IgG conjugated with horseradish peroxidase (Bio-Rad, Hercules, CA, USA) was served as the secondary antibody at a 1:10000 dilution.

### MS/MS analysis

The CBB-stained SDS-PAGE gel lane was cut out manually according to the results of the western blot coupled with analysis of the molecular weight of the target peptide (S1, S2 and S3 Figs). In-gel digestion was performed as previously described[18]. Briefly, the chopped gel was destained with 200–400 μL 100 mmol/L NH_4_HCO_3_ and 30% ACN and washed in milli-Q water. The supernatant was discarded. The resulting product was incubated at 25°C for 15 min with 100 mM NH_4_HCO_3_ and then dried in a SpeedVac (Thermo Savant) for 30 min. The dried gel particles were rehydrated at 4°C for 40 min with 2.5 μL/well trypsin (sequencing grade; Promega, Madison, WI) in 50 mM ammonium bicarbonate (20 μg/mL) and then incubated at 37°C overnight. The enzymatic solution was transferred out, and the remaining solution was sonicated for 15 min and lyophilized together with the enzymatic solution. The powder was dissolved in 1.5 μL of 0.1% trifluoroacetic acid for later use.

The prepared peptide mixtures were mixed with an equal volume of matrix solution (R-cyano-4-hydroxy-cinnamic acid (CHCA, Sigma, St. Louis, MO) in 0.1% TFA and 50% ACN) and spotted on the target plate. Samples were allowed to air-dry and analyzed using a 5800 MALDI-TOF/TOF Proteomics Analyzer (Applied Biosystems, Foster City, CA, USA). The MASCOT search engine (version 2.2; Matrix Science, London, UK) was used for peptide and protein identification with the following parameters: taxonomy of all entries, trypsin digestion with one missing cleavage, fixed modifications of carbamidomethyl, dynamical modifications of oxidation, peptide mass tolerance: ± 100 ppm, fragments mass tolerance: ± 0.4 Da. The search database includes two sections: (1) the 20806 sequences from NCBI_*Bombyx*, download on 07/23/2013; (2) TEST database, including the polypeptide corresponding to the calcium-binding domain at the 3’-flank of the silk light chain: CABP-FLpolyA. The criterion of a successful identification is that the protein score C.I.%≥95% and total ion confirmed C.I.%≥95%.

### Ca-binding activity assay of transgenic silkworm silk fibroin

The Ca-binding activity of the silk fibroin of transgenic silkworm was detected by the methods described by Nagano et al. [12]. In brief, 1.5 ml of a 100 mM CaCl_2_ solution was added into a mixture of 1.5 ml of 100 mM NaHCO_3_ (pH 8.7) and 300 μl of the transgenic silk fibroin (10 mg/ml). The precipitation of CaCO_3_ from the mixture of CaCl_2_ and NaHCO_3_ solution was prevented by the presence of transgenic silkworm silk fibroin. The rate of CaCO_3_ precipitate formation can be monitored as the adsorption at 570 nm using a V-530 type UV–vis spectrometer (Jasco Inc., Japan) because CaCO_3_ has an absorption maximum at 570 nm. Fresh NaHCO_3_ solution (pH 8.7) was prepared to maintain the pH of the solution at 8.7. The Ca-binding activity of native silk fibroin was also examined under the same experimental conditions.

### Mechanical properties and thermal behavior measurement of silk of transgenic silkworm

The mechanical properties of the specimens were measured at a crosshead speed of 2 mm/min using an Instron testing frame (AGS-J, Shimadzu, Japan) with a 10 N capacity load cell. Four parallel samples were taken, and five single silks were combined into one sample[20]. The thermal behavior of the samples was analyzed using DSC (Mettler Toledo, Netherlands). All of the samples were ground into powder and then loaded in an aluminum crucible under dry conditions. DSC measurements were performed in a nitrogen atmosphere with temperature ranging from 30 to 500°C at a heating rate of 10°C/min[21].

## Results

### Generation of transgenic silkworms

**Fig 2:**
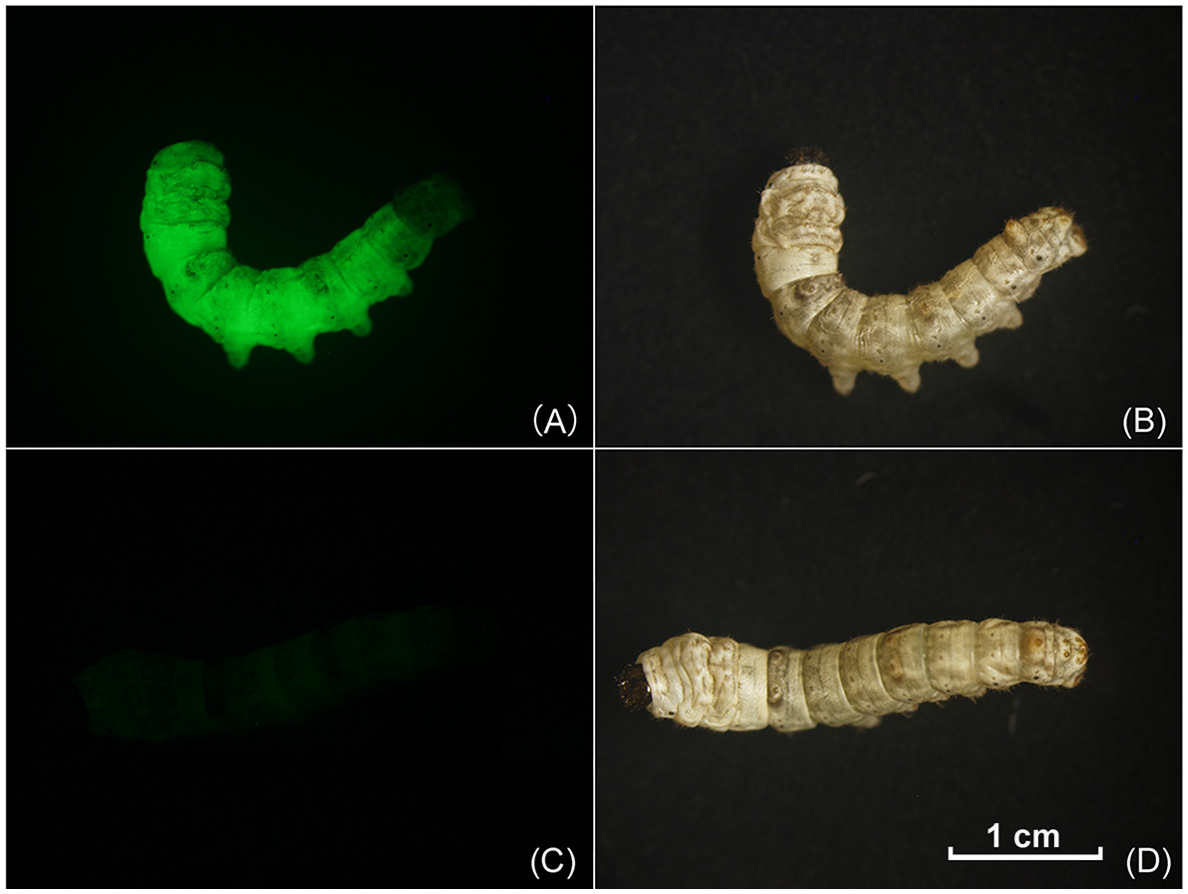
G1 transgenic silkworms positive for EGFP. Transformed larvae are fluorescent compared with non-transformed controls. The transgenic silkworm under fluorescence (A) and visible light (B). The native silkworm Lan 10 under fluorescence (C) and visible light (D).

To produce the silks that possess a high Ca-binding activity, a transgenic silkworm that produces recombinant silk with the 3’-flank of the fibroin light chain was constructed. A transformation vector, pBCABP-EGFP, was designed to produce recombinant fibroin (Fig 1b). In the transgenic experiment, the pBCABP-EGFP vector was injected into 1200 eggs of the Lan10 strain, and transgenic silkworms were screened for the expression of GFP in the whole body of G1 silkworms at the 1^st^ instar under a fluorescence microscope equipped with a filter set for EGFP (Fig 2). According to the screening results of EGFP, there were 8 positive broods at G1, and the transgenic efficiency was 21.62% (Table 1). In the 8 positive broods, the numbers of positive individuals were different: 7, 10, 9, 6, 14, 21, 6 and 4. According to a study in our lab, EGFP expression is related to the expression of exogenous genes contiguous to it [22]. Based on the expression characteristics of the reporter gene EGFP, 17 G2 families were separated from 8 G1 positive broods and were named SCa1–SCa17.

**Table 1:** The efficiency of the transgenic experiment.

Strain Name	Injected eggs	Examined larvae at G0	Hatchability (%)	Moths at G0	Survival rate (%)	EGFP- positive larvae at G0	Positive probability (%)
Lan10	1200	116	9.67	37	31.9	8	21.62

### Integration site analysis of transgenic silkworms

A foreign gene at different genomic loci results in diverse expression levels due to position effects. The families SCa3, SCa8 and SCa10 were chose to measure the impact of the insertion site on the Ca-binding activity of silk. The foreign gene expression vectors of SCa3 and SCa8 mediated by *piggyBac* transposon were inserted into Bm-scaf2, at positions 4272208 to 4272100 of chromosome 18, and the vector of SCa10 was inserted into Bm-scaf4, at positions 1155464 to 1155065 of chromosome 16 (S1 Table).

### Identification results by western blot

**Fig 3:**
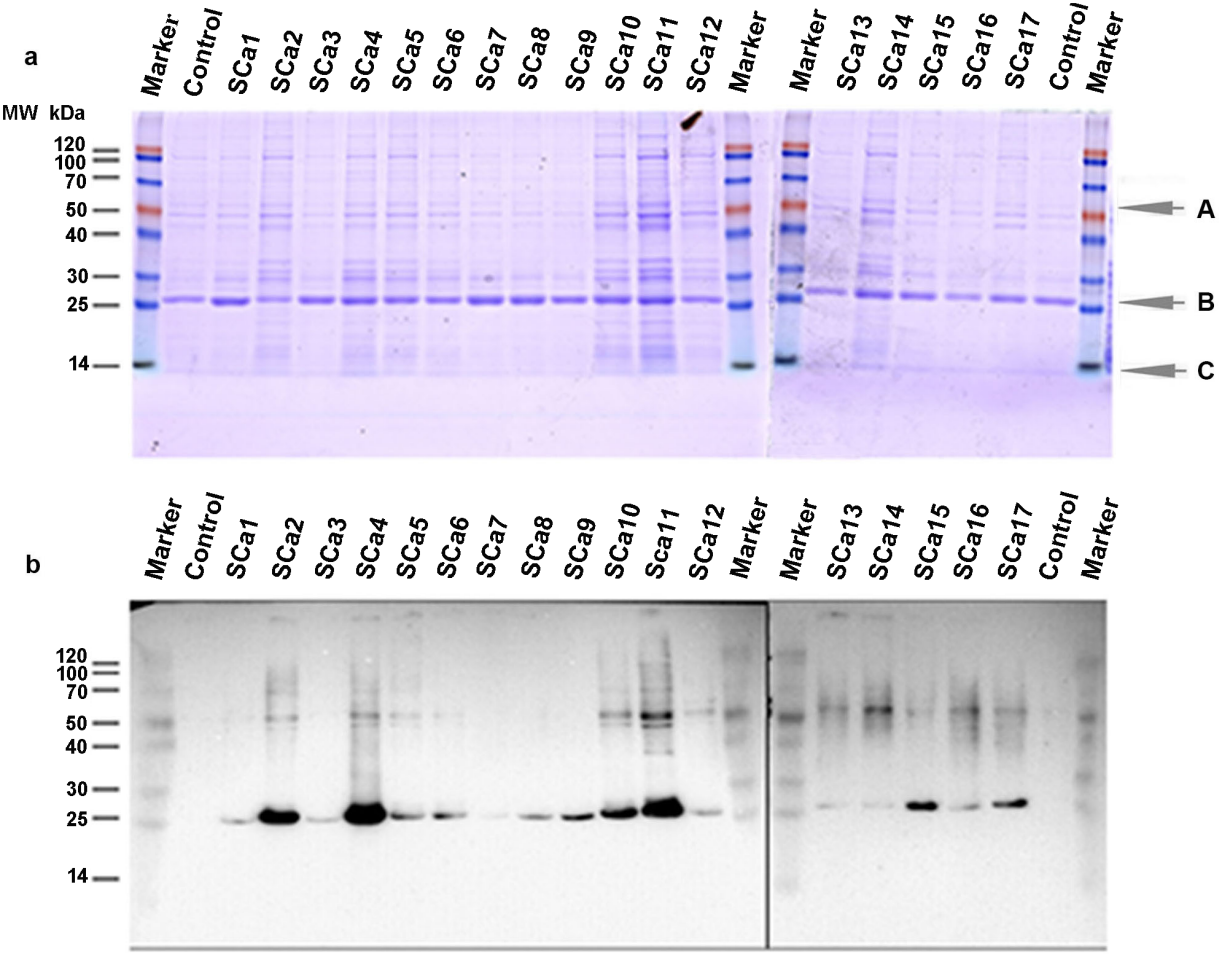
Western blot analysis of cocoon silk. The SDS-PAGE identification of cocoon silk from 17 transgenic families (A) and their corresponding western blot results (B). The antibody was diluted to 1: 2000. The protein marker is shown at the left of the figure as M; Ct: Control strain Lan 10; SCa1-SCa17: transgenic family. A, B, C indicate three protein bands with different molecular weights. The corresponding bands were cut out for MS/MS identification.

The expression levels of the Ca-binding protein in the transgenic silk fibroin of 17 families, SCa1–SCa17, were identified using the western blot method. Donor strain lan10 was used as the transgenic control. The results showed that there were no hybridizing bands identified in either type of sample, posterior silk glands and silk fibroin, at the target band molecular weight of 12.1 kDa. However, existing specific bands may appear at approximately 50 kDa and 25 kDa were identified in all 17 of these families (Fig 3, S4 Fig), but they were not identified in the control strain. We can infer from these results that the Ca-binding protein was expressed in the posterior silk gland and secreted into the silk fibroin. The Ca-binding protein may have appeared as a dimer.

### Identification results of MS/MS

The bands identified by western blot analysis were further identified by an MS/MS experiment. The results showed that one protein, UDP-glycosyl transferase UGT34A2 of *Bombyx mori*, was identified at band A (Fig 3; S2 Table, S1 Fig); two proteins were identified at band B, fibroin light chain and Ca-binding protein (Fig 3; S3 Table, S2 Fig); the fibroin light chain was identified at band C (Fig 3; S4 Table, S3 Fig). Both western blot and MS/MS analysis further illustrate that band B is Ca-binding protein, and the Ca-binding protein exists in the cells of silk glands as dimer.

### Calcium binding of transgenic silk fibroin

**Fig 4:**
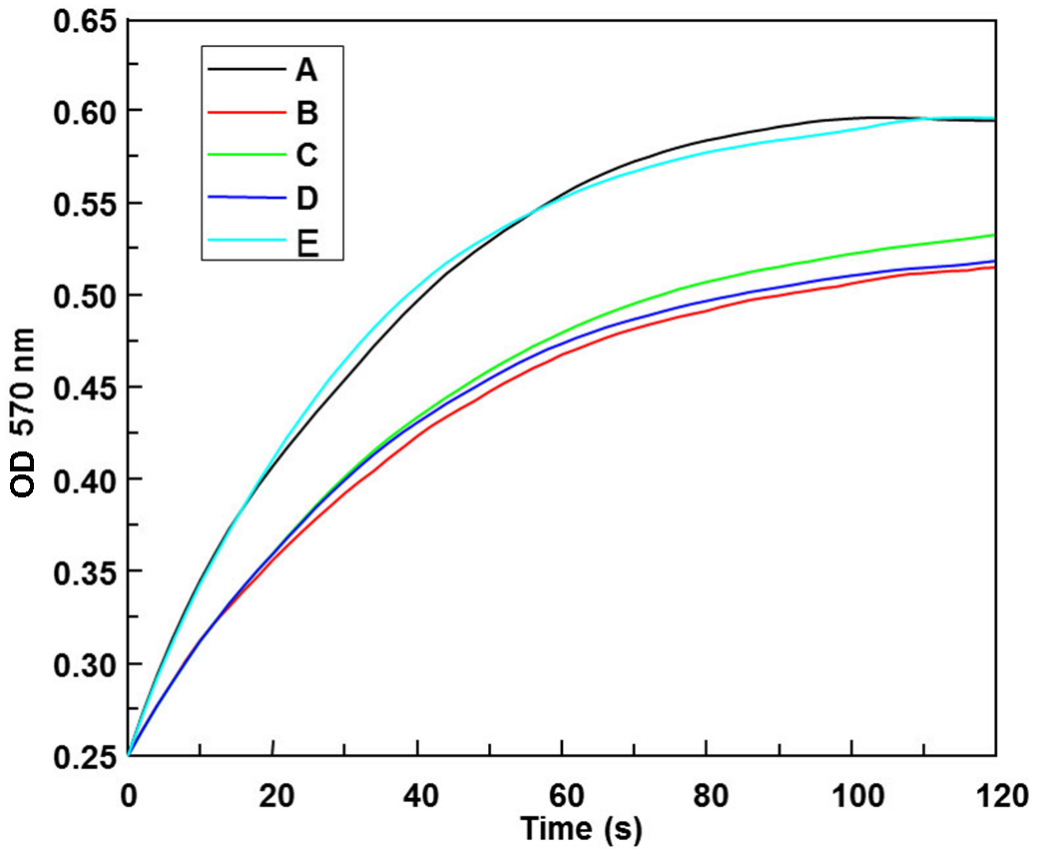
The Ca-binding activity measurement of the fibroin solution. Black line, CaCl_2_ control (A), red line, natural silk (B), green line, transgenic silkworm SCa3 (C), blue line, transgenic silkworm SCa8 (D), light blue line, transgenic silkworm SCa10 (E).

The Ca-binding activity of silk fibroin from SCa3, SCa8, and SCa10 was measured (Fig 4). The inhibitory effect on the production of CaCO_3_ is higher for transgenic silk fibroin compared with non-transgenic silk fibroin. In particular, the transgenic strain Sca10 showed significantly higher Ca-binding activity than SCa3 and SCa8. This result was expected due to the presence of the Ca-binding sequence EYDYDDDSDDDDEWD from the protein MSI60 in the silk fibroin.

### The mechanical properties and thermal characteristics of transgenic silk fibroin

**Fig 5:**
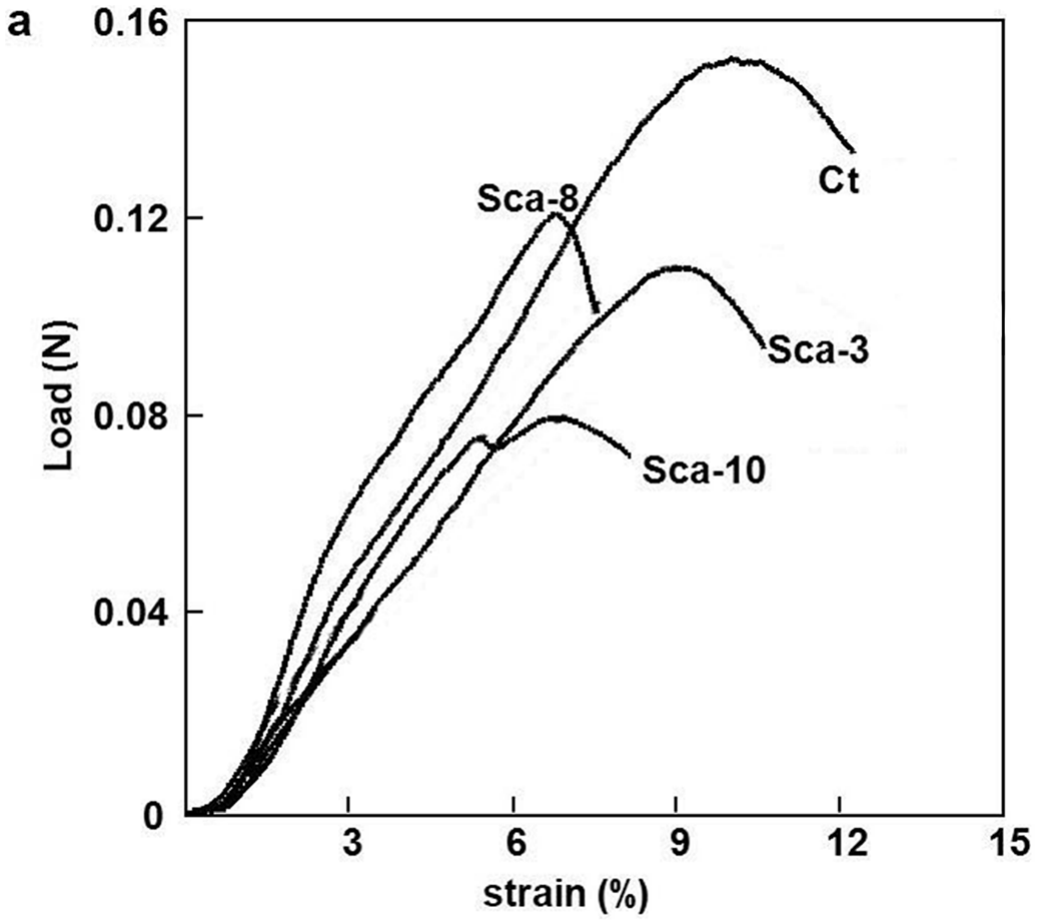
The detection of the mechanical properties of composite silk fibers. (A) The measurement of the load (N) of the transgenic silk. (B) The measurement of the relative tensile modulus.

**Fig 6:**
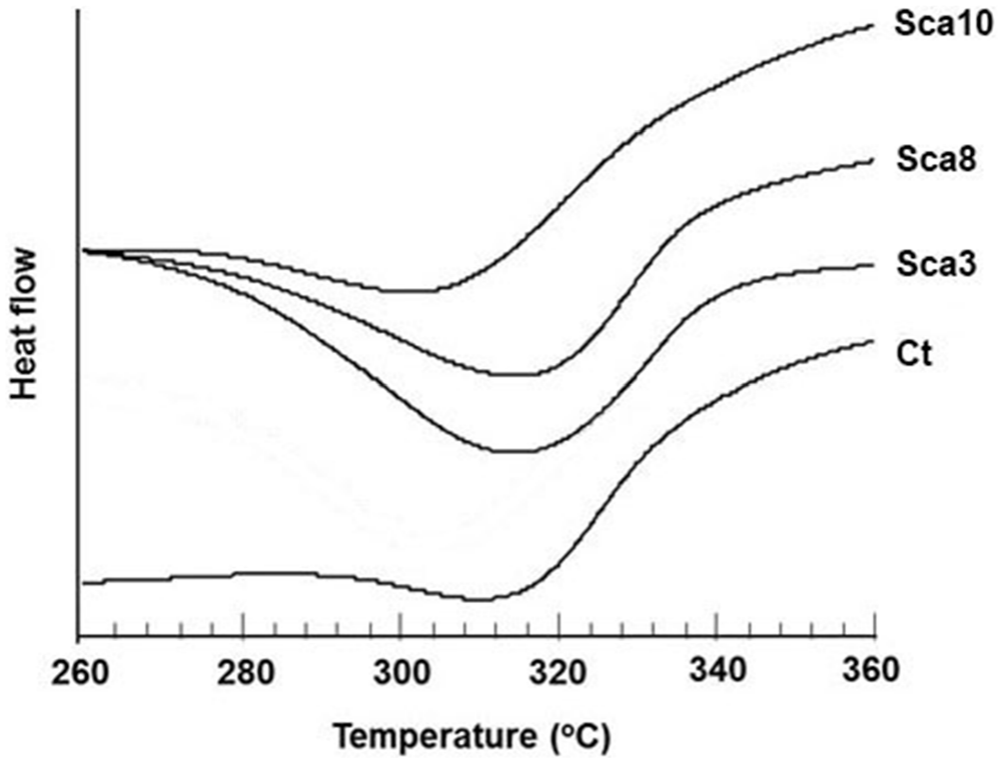
The measurement of the thermal characteristics of the transgenic silk. The exothermic peaks of Sca3, Sca8 and Sca10 appear at 314, 314 and 303°C, respectively.

The mechanical properties of composite silk fibers produced by the silkworm families SCa3, SCa8, and SCa10 were measured in parallel under precisely matched conditions. The control group showed the highest tensile stress and elongation at breaking (Fig 5). The mechanical properties of the transgenic lines declined slightly compared to the unmodified strain but were similar among themselves. The thermal characteristics of the transgenic silk were also identified (Fig 6). The transgenic silk of Sca3, Sca8 and Sca10 presented exothermic peaks at 314°C, 314°C and 303°C, respectively, followed by the degradation of silk fibroin. In contrast, the exothermic peak of native silk appears at 310°C. There was no endothermic peak or random coil from SILKIto SILKIIobserved for any of the silk families that showed the high crystal properties of silk fiber. These results showed that CABP will change the crystallization level of silk and alter its mechanical properties.

## Discussion

The sequence [(AGSGAG)_6_ASEYDYDDDSDDDDEWD]_2_ from the shell nacreous matrix protein MSI60[15] was used to generate silk fibroin with Ca-binding activity. Cast films made from silk-like peptides with the MSI60 domain are indeed able to bind calcium ions[14]. A predominantly*β*-sheet silk protein, *Bombyx mori* fibroin, has considerable potential use in strong and tough implantable biomaterials and scaffolds for tissue engineering due to its mechanical properties, biocompatibility, and restorability[23]. The capability of mineralization of natural silk fibroins with the bone mineral hydroxyapatite indicates that silk fibroins may have potential use as bone graft substitute materials[24].

In our study, SCa3 and SCa8 were found to have the same insertion site. To examine the impact of the integration site on the expression level of exogenous proteins, families SCa3, SCa8 and SCa10 were selected to analyze the activity of the Ca-binding protein. The foreign protein abundance is different because of the different insertion sites[22]. The Ca-binding activity, mechanical properties and thermal characteristics of transgenic silk fibroin of families SCa3 and SCa8 are similar, but those of SCa10 are different. This result also can be seen from the western blot experiment in which the different transgenic families were shown to have different expression levels of Ca-binding protein (Fig 3, S4 Fig). These results further confirmed that the *piggyBac* transposon-mediated transformation resulted in random insertions into the genome and that foreign gene transcription is dramatically influenced by the position effect.

The inhibitory effect of the transgenic silk fibroin on the production of CaCO_3_ is higher compared with native silk fibroin (Fig 4), which indicated that the Ca-binding activity of silk fibroin from SCa3, SCa8 and SCa10 is higher than that of the silk from non-transgenic families. At the same time, the mechanical properties and thermal characteristics of transgenic silk fibroin did not decrease significantly (Figs 5 and 6). These excellent features will facilitate the practical application of transgenic silk with calcium-binding activity.

## Supporting Information

S1 Fig The MS (A) and MS/MS (B) map of the proteins from band A of Fig 3. (TIF)

S2 Fig The MS (A) and MS/MS (B) map of the proteins from band B of Fig 3. (TIF)

S3 Fig The MS (A) and MS/MS (B) map of the proteins from band C of Fig 3. (TIF)

S4 Fig Western blot analysis of posterior silk gland. The SDS-PAGE identification of posterior silk gland from 17 transgenic families (a) and their corresponding western blot results. The antibody was diluted to 1:2000 (b). The protein marker is shown at the left of the figure as M; Ct: Control strain; SCa1–SCa17: transgenic families.
(TIF)

S1 Table The sequences of the insertion sites and their locations in the genome. (DOC)

S2 Table The MS/MS identification results of band A in Fig 3. (XLSX)

S3 Table The MS/MS identification results of band B in Fig 3. (XLSX)

S4 Table The MS/MS identification results of point C in Fig 3. (XLSX)
