# Supplementary figures and images for: Characterization of Transgenic Silkworm Yielded Biomaterials with Calcium-Binding Activity
Source: PLoS One. 2016 Jul 14;11(7):e0159111. doi: 10.1371/journal.pone.0159111 (PMC4944971; doi:10.1371/journal.pone.0159111)

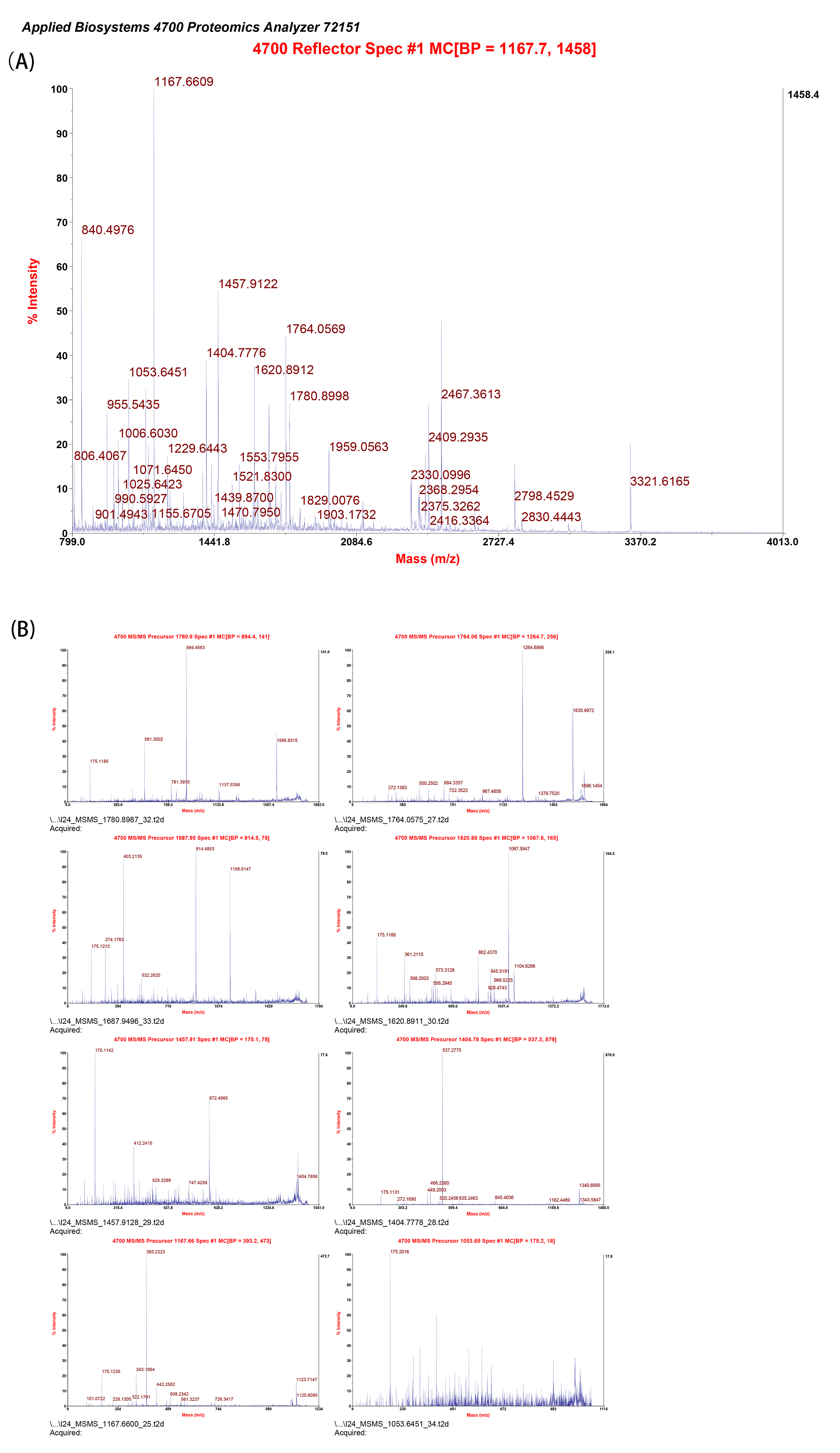

Supplement: S1 Fig — (TIF) [file pone.0159111.s001.tif]

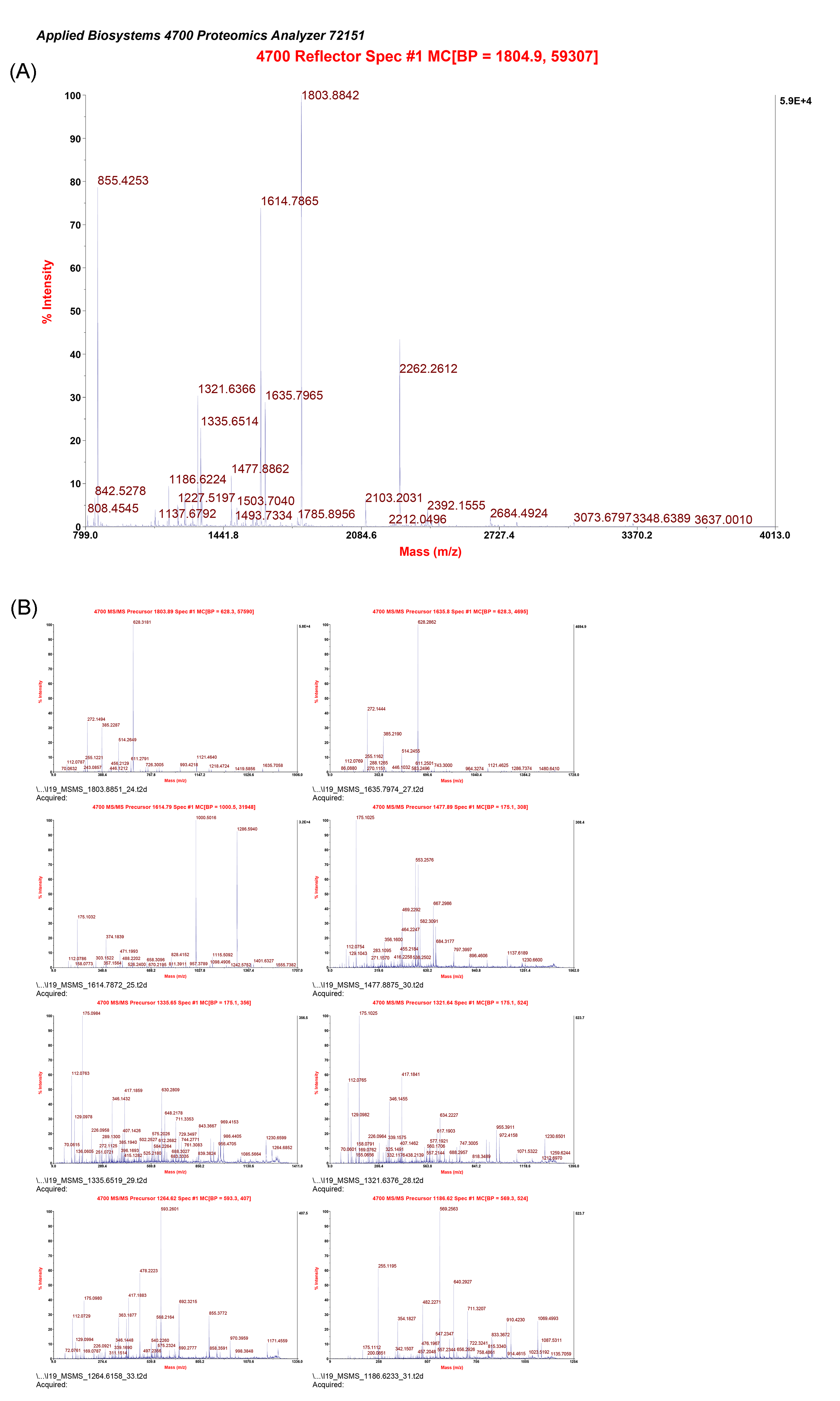

Supplement: S2 Fig — (TIF) [file pone.0159111.s002.tif]

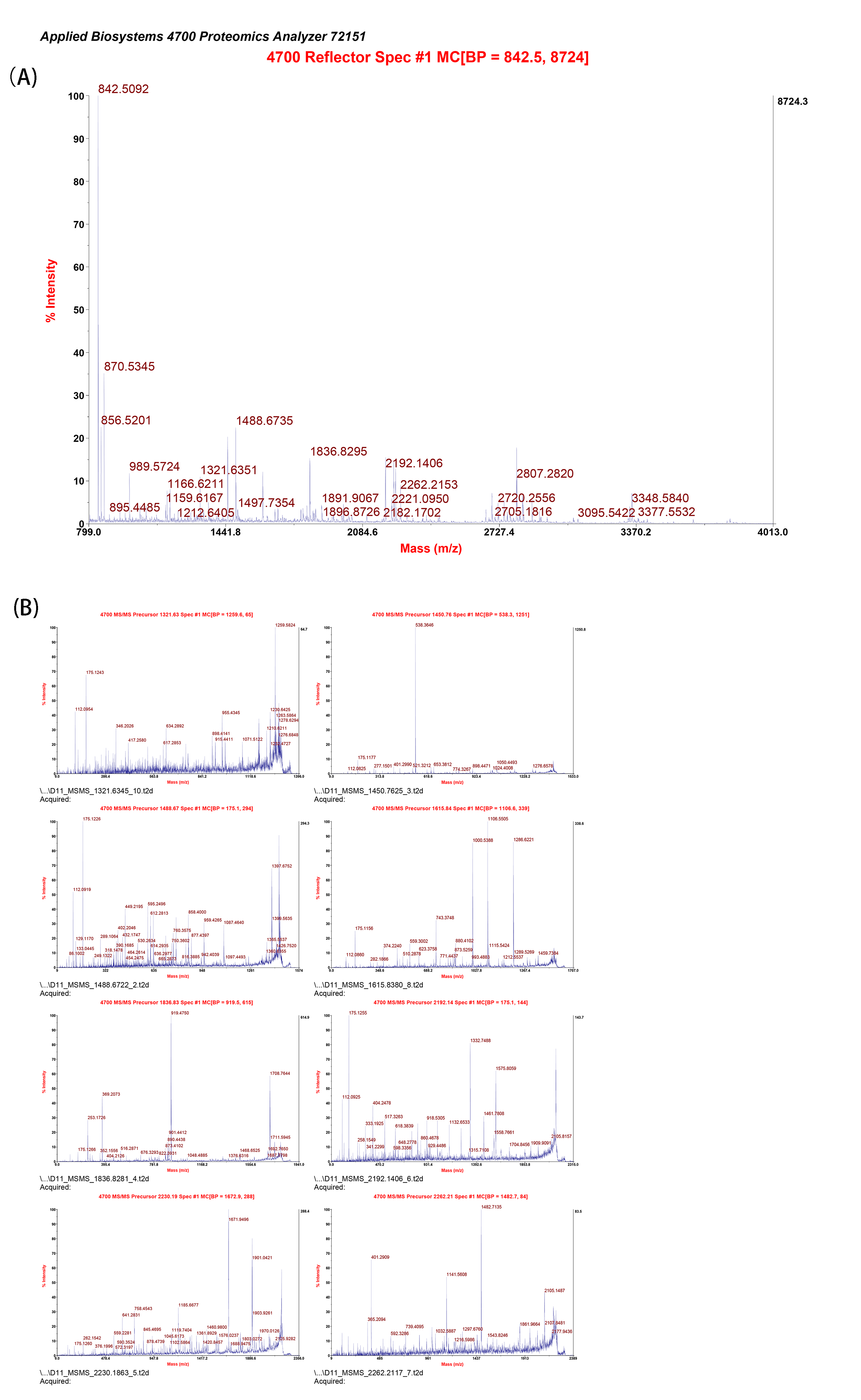

Supplement: S3 Fig — (TIF) [file pone.0159111.s003.tif]

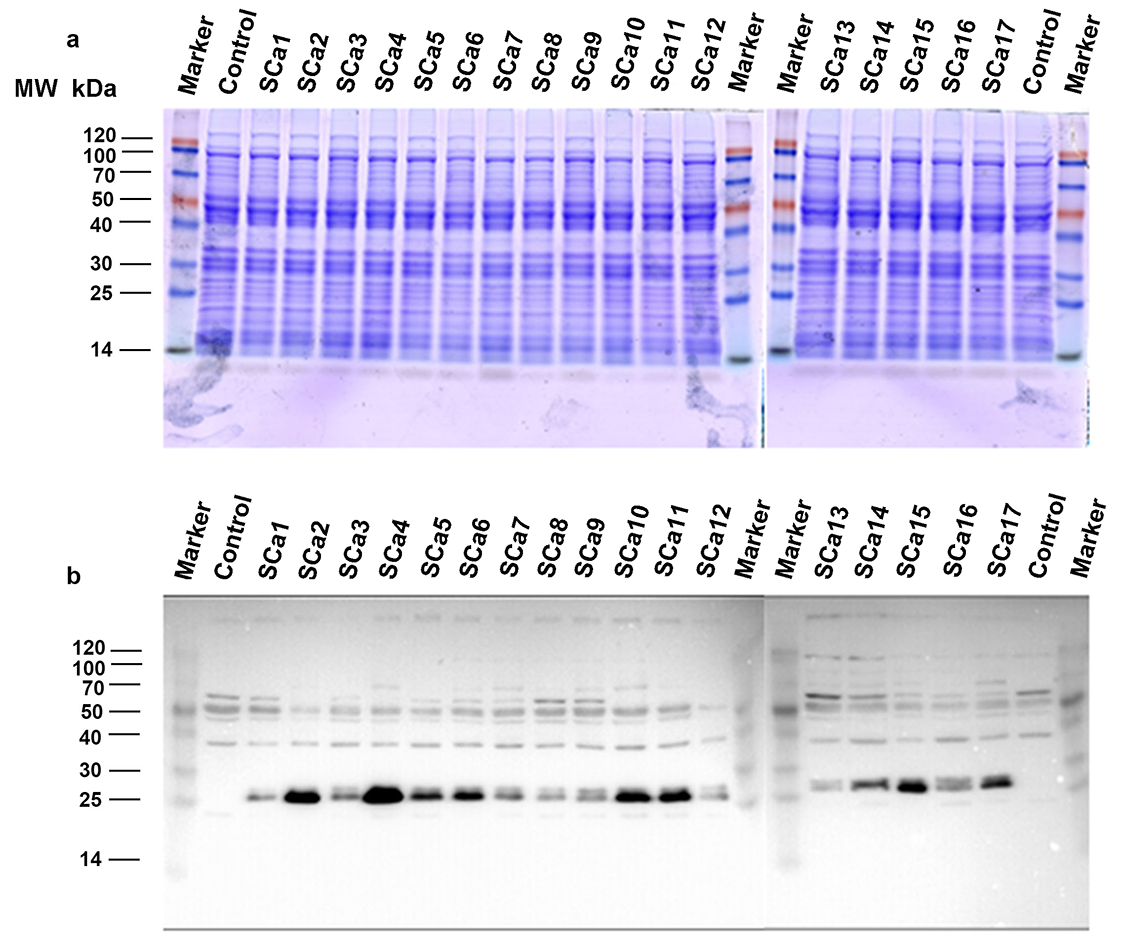

Supplement: S4 Fig — The SDS-PAGE identification of posterior silk gland from 17 transgenic families (a) and their corresponding western blot results. The antibody was diluted to 1:2000 (b). The protein marker is shown at the left of the figure as M; Ct: Control strain; SCa1–SCa17: transgenic families. (TIF) [file pone.0159111.s004.tif]
