# Supplementary material for: Characterization of Transgenic Silkworm Yielded Biomaterials with Calcium-Binding Activity
Source: PLoS One. 2016 Jul 14;11(7):e0159111. doi: 10.1371/journal.pone.0159111 (PMC4944971; doi:10.1371/journal.pone.0159111)
Supplement: S1 Table — (DOC) [file pone.0159111.s005.doc]

**S1 table.** The sequences of the insert site and their locations on the genome.

| Transgenic family | Hit scaffold ID | Insert site | Insert region | sequences |
| --- | --- | --- | --- | --- |
| Sca3 | Bm_scaf2 | chr18:4272208-4272100 | intron | AAAAGGAAAATTTCAATTAATTTTGTTAATTAACTATAGCAAGTCCATGTTTCTGAAGGAATACCGTGAAATGAGTTTATCCAGAACGCTTTGAGATAAAC |
| Sca8 | Bm_scaf2 | chr18:4272096-4272208 | intron | TTAAGTTTATCTCAAAGCGTTCTGGATAAACTCATTTCACGGTATTCCTTCAGAAACATGGACTTGCTATAGTTAATTAACAAAATTAATTGAAATTTTCCTTTTGATCCGAATGACAACTTTAGTTTTTAATGTTATGCATATAGGCATTCATAAAGCCAAATAAATATTCACGTCATAAAATTTTAAACTACCGTCATTA |
| Sca10 | Bm_scaf4 | chr16:1155464-1155065 | intron | TTGGTATCCACCAAAACTTGTACGCCAGGTATCCGGCTGCTGCTAGGCTCAGGATTATGTACAGAGACACTGATGACCAGCTTGGATTGTTGGTTATTATTGTCCTGATATTTTCAGTATTTTCCTGGATTTTTTCAGCGCGTTGCAGGGCGTCTTTAAGTTTATCCAGTTTAATGTCTTGTAACTTGATGTTTATTGGAGCTGCTTCGGGTGTAGCATGTGCACGGGGTAAAGGTATTATTTCTTCAATGTTTATGAATTTTCAGTGTGTTTGAAGGGTTTTTCCAGCGACTTGTACCTTACAATTATTGTCTATTGTAATTAGGGAAATGCCTTTGCTACGCTGGTACTGCACTTCATTGTTGCAGGTTGACTTGATAACTTCTTCTTGT |
